# The Elg1 Clamp Loader Plays a Role in Sister Chromatid Cohesion

**DOI:** 10.1371/journal.pone.0005497

**Published:** 2009-05-11

**Authors:** Oren Parnas, Adi Zipin-Roitman, Yuval Mazor, Batia Liefshitz, Shay Ben-Aroya, Martin Kupiec

**Affiliations:** 1 Department of Molecular Microbiology and Biotechnology, Tel Aviv University, Ramat Aviv, Israel; 2 Michael Smith Laboratories, University of British Columbia, Vancouver, British Columbia, Canada; Pasteur Institute, France

## Abstract

Mutations in the *ELG1* gene of yeast lead to genomic instability, manifested in high levels of genetic recombination, chromosome loss, and gross chromosomal rearrangements. Elg1 shows similarity to the large subunit of the Replication Factor C clamp loader, and forms a RFC-like (RLC) complex in conjunction with the 4 small RFC subunits. Two additional RLCs exist in yeast: in one of them the large subunit is Ctf18, and in the other, Rad24. Ctf18 has been characterized as the RLC that functions in sister chromatid cohesion. Here we present evidence that the Elg1 RLC (but not Rad24) also plays an important role in this process. A genetic screen identified the cohesin subunit Mcd1/Scc1 and its loader Scc2 as suppressors of the synthetic lethality between *elg1* and *ctf4*. We describe genetic interactions between *ELG1* and genes encoding cohesin subunits and their accessory proteins. We also show that defects in Elg1 lead to higher precocious sister chromatid separation, and that Ctf18 and Elg1 affect cohesion via a joint pathway. Finally, we localize both Ctf18 and Elg1 to chromatin and show that Elg1 plays a role in the recruitment of Ctf18. Our results suggest that Elg1, Ctf4, and Ctf18 may coordinate the relative movement of the replication fork with respect to the cohesin ring.

## Introduction

Genomic instability is a hallmark of cancer cells. Most human cancer cells show signs of genome instability, ranging from elevated mutation rates, to gross chromosomal rearrangements including deletions and translocations. Several surveillance and repair mechanisms operate in eukaryotic cells to ensure the stability of the genome. The current view is that most spontaneous chromosomal rearrangements arise during DNA replication. The activity of the DNA polymerases may be impaired by the presence of secondary structures, bound proteins or DNA lesions; this may lead to stalling or even collapse of replication forks. In response, cellular mechanisms are activated that arrest cell cycle progression, induce DNA repair, and restore replication [1], [2]. These repair mechanisms act on lesions to promote their repair and to prevent them from being converted into fatal genomic rearrangements. Pathways for DNA repair and genome stability are highly conserved across species. Because of this conservation, simple organisms, such as the budding yeast *Saccharomyces cerevisiae,* are extremely useful for studying the basic principles of genome stability and maintenance.

The *ELG1* gene was identified in yeast as a mutant that causes enhanced levels of genomic instability [3], [4], [5]. Deletion of *ELG1* in yeast leads to increased recombination levels [4], [6], as well as elevated levels of chromosome loss [4], [7] and gross chromosomal rearrangements [7]. *elg1* mutants also exhibit elongated telomeres [8] and increased levels of Ty transposition [9]. Elg1 function is thus clearly required for maintaining genome stability during normal growth, and its absence has severe genetic consequences. The Elg1 protein shares sequence homology with the large subunit of RFC clamp loader and with the product of two additional genes involved in checkpoint functions and genome maintenance: *RAD24* and *CTF18*. Elg1, Rad24 and Ctf18 form three alternative RFC-like (RLC) protein complexes, by interacting with the four small subunits of RFC [3], [4], [5]. The Rad24 RLC (Rad17 in *S. pombe* and hRad17 humans) [10] is predicted to load an alternative DNA sliding clamp (9-1-1) composed of checkpoint proteins [Ddc1/Rad17/Mec3 in *S. cerevisiae*, Rad9/Rad1/Hus1 in *S. pombe* and humans] [11]. Genetic data indicates that the Elg1, Ctf18 and Rad24 RLCs work in three separate pathways important for maintaining the integrity of the genome and for coping with various genomic stresses. Mutations in each of the genes cause a mild sensitivity to DNA damage or DNA replication block. Double mutants show increased sensitivity, whereas the triple mutant is extremely sensitive and shows impaired growth [4].

Genetic screens in yeast have shown that *elg1* mutants have synthetic growth defects when combined with genes that are involved in homologous recombination, DNA repair, replication fork restart, checkpoint response and sister chromatid cohesion [2], [3], [12]. These genetic interactions emphasize the pivotal role of Elg1 as a guardian of genomic stability. Among the strongest synthetic interactions is the one with *ctf4*: double mutant spores *elg1 ctf4* germinate, but fail to undergo cell divisions [3], [4], [13]. In addition, Δ*ctf4* and Δ*elg1* mutant cells show partially overlapping phenotypes. Both mutants have high levels of recombination, chromosome loss, as well as synthetic growth defects with *rad52*, *mec1* [14], [15] and other genes that are involved in DNA metabolism.

*CTF4* was found, together with *CTF18,* in two independent screens for mutants that affect chromosome transmission fidelity [16], [17]. Both *ctf4* and *ctf18* exhibit elevated levels of recombination [18] and share genetic interactions with genes involved in DNA replication [14]. The proposed role for these proteins is in sister chromatid cohesion [19], [20]. Sister chromatids are held close to each other with the help of a complex called cohesin. Cohesin is composed of four subunits and forms a ring, although the precise topology of the ring relative to the replicated DNA is still debated [e.g.: [21], [22]]. Cohesin complexes are loaded on the DNA during late G1 at specific sites along the chromosomes. Although they are present along the whole chromosome arm with an average distance of 11 kb, they are especially enriched near the centromeres [23]. Sister chromatids remain associated from the beginning of S phase throughout G2, until metaphase. During mitosis the microtubules are bound to the kinetochore, establishing bipolar attachment to the spindle apparatus. Cohesin complexes oppose the tension that is implemented by the microtubules until all the chromosomes are bound to the microtubules properly and are arranged in the metaphase plate. Only then, a tightly regulated cascade is activated to trigger the cleavage of Mcd1/Scc1 (a cohesin subunit, hereafter referred to as Scc1 for simplicity), enabling the separation of sister chromosomes to opposite poles of the nucleus. Defects in cohesin loading, establishment, maintenance or cleavage lead to chromosome loss and cell death.

A clear linkage exists between replication progression and sister chromatid cohesion. First, Ctf7/Eco1, encoded by another gene that was found in the *ctf* screen for increased chromosome loss [17], is required for cohesion establishment solely during S phase. Mutations in this gene are synthetic lethal (SL) with mutations in the DNA replication clamp PCNA and overexpression of PCNA partially suppresses temperature sensitive *ctf7* mutants, allowing them to grow at a higher temperature before arresting [24], [25], [26]. Second, loading of cohesin at the beginning of S phase is essential for proper sister chromatid cohesion. If cohesin subunits are expressed after DNA replication, they are successfully loaded onto the chromatin but fail to establish sister chromatid cohesion [27]. Finally, in the absence of Ctf4 or Ctf18, which are proteins that act close to the replication fork [28], a strong phenotype of precocious sister chromatid separation is observed. In addition, Ctf4 was shown to bind physically to the catalytic subunit of polymerase α [14].

Although *ELG1* has tight genetic interactions with *CTF4* and *CTF18,* it is not known how it affects genomic stability. Here we performed a genetic screen in order to find high copy number suppressors of the SL interaction between *elg1* and *ctf4*. We found a cohesin subunit and a cohesin loader, pointing to the fact that the synergistic genetic interaction between *elg1* and *ctf4* is due to enhanced defects in sister chromatid cohesion. Consistent with this possibility we also show that *elg1* mutants exhibit defects in cohesion. Our results show that both the Elg1 and the Ctf18 clamp loaders affect sister chromatin cohesion and establish a link between Elg1 activity and cohesin loading.

## Results

### A screen for high copy number suppressors of the synthetic lethal phenotype of *elg1 ctf4*

**Figure 1:**
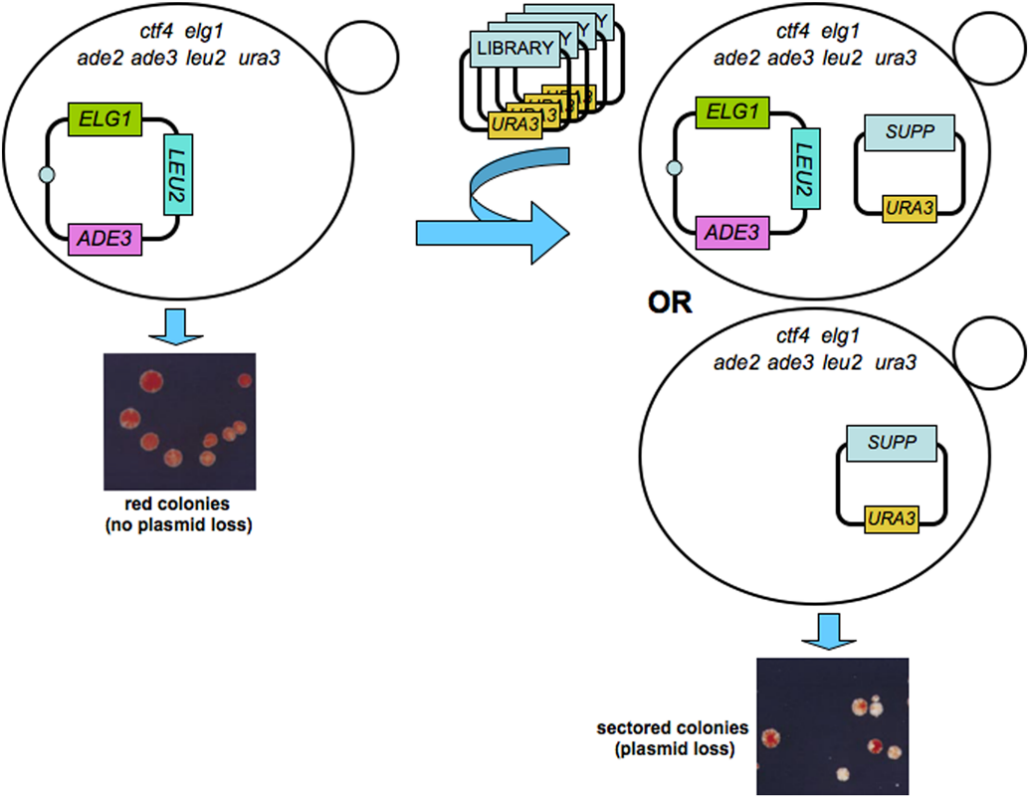
Schematic explanation of the genetic screen carried out. A double mutant *elg1 ctf4* strain is kept alive by the presence of a plasmid carrying the *ELG1* gene. The *ADE3* marker on the plasmid confers a red pigment to the cells carrying it. As these cells are unable to lose the plasmid during colony formation, all colonies are uniformly red. This strain was transformed with a high copy number library carrying random fragments of the yeast genome. Cells that received a plasmid that suppresses the synthetic lethality phenotype can now lose the *ELG1*-containing plasmid, becoming white. These cells create white or red/white sectored colonies.

Mutations in *ELG1* and *CTF4* are synthetic lethal (SL), namely, despite the fact that each single-mutant grows well, the double mutant is inviable [2], [4]. In order to further investigate the interaction between these genes, we carried out a genetic screen for high-copy-number suppressors of the synthetic lethality (Figure 1). First, we constructed a double mutant strain carrying a *ELG1* centromeric plasmid enabling the strain to stay alive. This plasmid has two marker genes: *LEU2* and *ADE3*. In the appropriate genetic background, the presence of the *ADE3* gene causes accumulation of a red pigment. Thus, *elg1 ctf4* strains carrying the *LEU2/ADE3/ELG1* plasmid grow well, and form uniformly red colonies, as they are unable to lose the *ELG1*-containing plasmid. These cells were then transformed with a yeast genomic library cloned in a high copy number plasmid (containing the *URA3* marker). Cells that received a plasmid with a gene that, when over-expressed, can suppress the SL phenotype of *elg1 ctf4* are now able to lose the *ELG1-ADE3-LEU2* plasmid, and therefore show white/red sectored colonies (Figure 1).

After transformation with the high copy number library, we screened about 30,000 colonies; 62 of them showed some degree of sectoring and were further analyzed. After re-introduction of the plasmid to naïve yeast strains, only 29 clones gave a positive result. Twenty six carried the *CTF4* gene, two clones carried a genomic DNA fragment containing the *SCC1* gene and an additional clone carried the *SCC2* gene. *SCC1* encodes a subunit of the Cohesin complex. This complex is composed of four core subunits: Smc1 and Smc3, which are members of the structural maintenance of chromosomes (SMC) protein family, and two non-SMC subunits, Scc1, which is a member of the kleisin family, and Scc3. Smc1, Smc3 and Scc1 form a ring large enough to contain two dsDNA molecules [21], [22]. Scc3 binds Scc1 and has an unknown function [27], [29]. The second gene isolated in our screen, *SCC2*, encodes a protein that forms, together with Scc4, a complex that helps loading cohesin on the DNA [30].

All the known phenotypes of mutants in *SCC1* are related to its function as a structural component of cohesin. This raises the question of whether Scc1 is the only subunit of the complex able to suppress the synthetic lethal phenotype of *elg1 ctf4* strains. The fact that *CTF4* was isolated numerous times but *ELG1* was not, suggest that the screen may not have been saturated enough. Alternatively, only Scc1 and Scc2 may be the limiting factors that determine the amount of cohesin complexes loaded onto the DNA. Scc1 may be the limiting component in cohesin formation, and Scc2 availability may be limiting the amount of Scc1 loaded. According to this possibility *ctf4 elg1* strain is inviable due to a low level of the cohesin ring, for example, at the centromeres.

**Figure 2:**
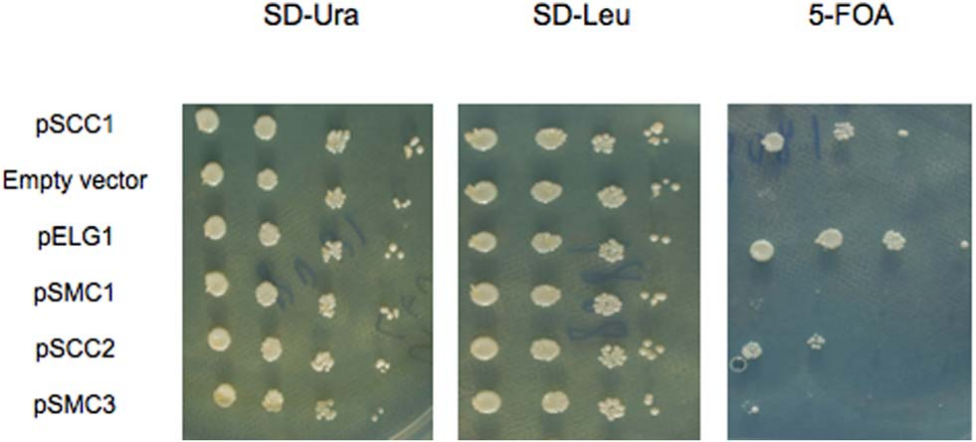
Overexpression of *SCC1* or *SCC2* suppress the synthetic lethality between *elg1* and *ctf4*. A *elg1 ctf4* double mutant maintained alive by the presence of a *URA3*-marked plasmid carrying the *ELG1* gene was transformed with *LEU2*-marked plasmids overexpressing *SCC1, SCC2, SMC1* or *SMC3,* or, as controls, with an empty vector or a plasmid carrying *ELG1*. Cells were successively diluted ten-fold and plated on plates lacking Uracil (SD-Ura), Leucine (SD-Leu), or on plates containing 5-fluoroorotic acid (5-FOA), which select for cells that became Ura- (i.e., lost the *URA3*-marked, *ELG1*-containing plasmid). Only plasmids overexpressing *SCC1* and *SCC2* allowed cells to grow on 5-FOA plates.

We therefore directly tested the ability of other cohesin subunits to suppress the SL phenotype by overexpressing *SCC1*, *SCC2, SMC1* or *SMC3* from *LEU2*-marked plasmids in a *ctf4 elg1* strain carrying a *ELG1*/*URA3* plasmid. If overexpression of any of these genes can suppress the SL phenotype, the *ELG1/URA3* plasmid can be lost, allowing the cells to grow on 5-FOA medium (which selects for Ura- cells). Figure 2 shows that *SCC1*and *SCC2*, (and of course *ELG1*), but not *SMC1* or *SMC3*, can rescue the SL phenotype of the *ctf4 elg1* double mutant strain. Thus, in agreement with the second hypothesis expressed above, Scc1 and Scc2 are the only limiting components able to suppress the lethality when overexpressed. Scc1 seems to be a slightly better suppressor than Scc2 (Figure 2).

One of the most prominent phenotypes of both *elg1* mutants and *ctf4* mutants is their increased levels of recombination. We tested whether the suppression of the SL phenotype by Scc1 overexpression was related to the hyper-recombination phenotypes observed. Table 1 shows that this was not the case, as overexpression of *SCC1* or *SCC2* failed to affect the hyper-recombination phenotype of either *elg1* or *ctf4* strains.

**Table 1:** Overexpression of Scc1 and Scc2 does not affect the hyper-recombination phenotype of *ctf4* or *elg1*.

Strain	His+ recombinants	Ty recombinants
MK166/vector	5.8×10^−6^ (×1)	1.1×10^−6^ (×1)
MK166/pSCC1	6.2×10^−6^ (×1.1)	1.2×10^−6^ (×1.1)
MK166/pSCC2	6.0×10^−6^ (×1.0)	0.97×10^−6^ (×0.9)
*elg1/*vector	30.6×10^−6^ (×5.3)	12.1×10^−6^ (×11)
*elg1/*pSCC1	37.7×10^−6^ (×6.5)	8.9×10^−6^ (×8)
*elg1/*pSCC2	33.1×10^−6^ (×5.7)	11.1×10^−6^ (×10)
*ctf4*/vector	20.2×10^−6^ (×3.5)	8.6×10^−6^ (×7.8)
*ctf4*/pSCC1	26.1×10^−6^ (×4.5)	9.1×10^−6^ (×8.3)
*ctf4*/pSCC2	22.1×10^−6^ (×3.8)	7.6×10^−6^ (×6.9)

**Figure 3:**
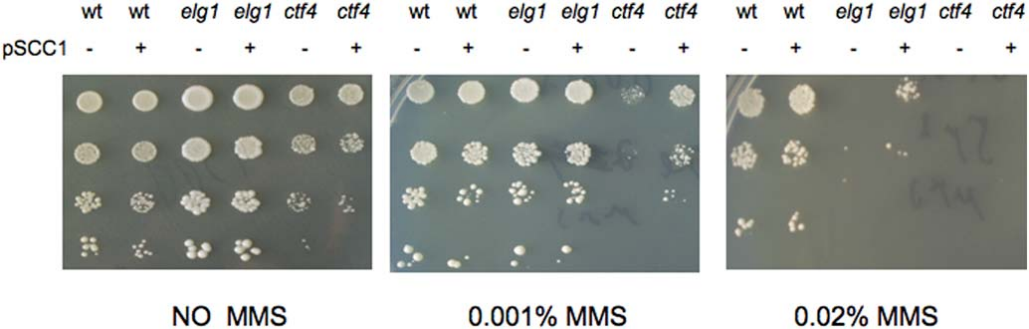
Overexpression of *SCC1* suppresses the sensitivity of both *elg1* and *ctf4* to DNA damaging agents. Isogenic wild type, *elg1* or *ctf4* strains were serially diluted and plated on plates without, and with methyl methanesulfonate (MMS) at two different concentrations (0.001% and 0.02%). Although *ctf4* strains are more sensitive than *elg1* strains, *SCC1* overexpression suppresses the sensitivity of both to MMS.

To investigate whether the suppression by Scc1 was due to bypass of the *elg1* phenotype, the *ctf4* phenotype, or the combination of both, we tested whether Scc1 overexpression could suppress the sensitivity of *elg1* or *ctf4* mutants to DNA damage. Figure 3 shows that Scc1 (and Scc2, data not shown) overexpression is able to partially suppress the sensitivity to MMS of both *ctf4* and *elg1* mutants, suggesting that the synthetic lethality, as well as the DNA damage sensitivity, are due to shared or overlapping functions involving sister chromatin cohesion. No changes in the cell cycle profile (as analyzed by FACS) were detected in these experiments (data not shown).

### Genetic interactions between *ELG1* and Cohesin subunits

**Figure 4:**
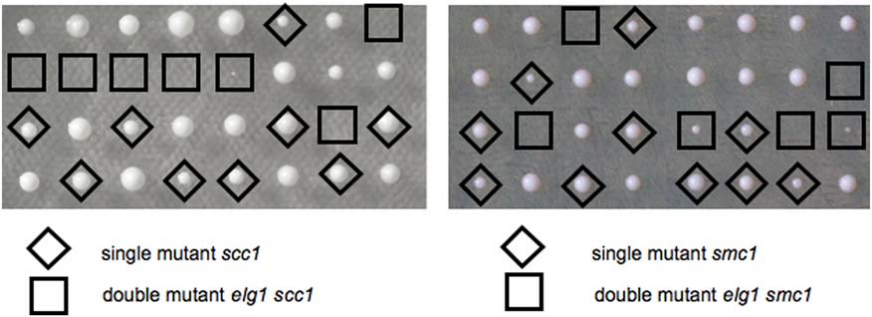
Mutations in *SCC1* and in *ELG1* show synthetic lethality. Tetrad analysis of double heterozygotes *elg1/ELG1 SCC1/scc1-73* and *elg1/ELG1 SMC1/smc1-259*. Plates were incubated at 25C, a permissive temperature for the single *scc1 and smc1* mutants. The double mutants were unable to form viable colonies.

In order to determine the genetic relationship between Elg1 and the Cohesin complex, we attempted to create double mutants between *elg1* and mutants defective in each of the components of cohesin. Figure 4 shows the results of tetrad dissection of *elg1/ELG1 SCC1/scc1-73* and *elg1/ELG1 SMC1/smc1-259* double heterozygous diploids. The double mutants *elg1 scc1* and *elg1 smc1* were very sick being either completely unable to form colonies or forming tiny, sick colonies that failed to develop.

We used the above method to examine the genetic relationship between *elg1* and mutations in other components of the cohesin complex such as Scc3 and Smc3, and additional genes that interact with cohesin, such as the loaders Scc2 and Scc4 [30], Esp1, the protease that cleaves Scc1 at the metaphase transition [31], Pds1, the securin that prevents cleavage until the right cell cycle stage is reached [32], and Pds5, a protein of unknown function recruited by cohesin and important for cohesion maintenance [33]. Results are presented in Table 2: *elg1* exhibited synthetic fitness phenotypes already at the permissive temperature when combined with any member of the cohesin complex or with the Scc2-Scc4 loaders, but not with mutations in the separin Esp1, in the securin Pds1 or in Pds5. These synthetic genetic interactions show that Elg1 activity is important in the early stages of cohesin establishment and not in the maintenance or the cleavage of cohesin, strengthening the possibility that both Elg1 and Ctf4 play an important role in the coordination between replication fork progression and cohesin loading.

**Table 2:** Synthetic genetic interactions between *elg1* and cohesin subunits/accessory factors.

Allele	Predicted Role	Synthetic effect with *elg1Δ*
*smc1-259*	Subunit of cohesin	Very sick
*smc3-42*	Subunit of cohesin	Very sick
*scc1-73*	Subunit of cohesin	Very sick
*scc3-1*	Subunit of cohesin	sick
*scc2-4*	Cohesin loader – acts during late G1	Very sick
*scc4-4*	Cohesin loader – forms a complex with Scc2	sick
*esp1-1*	The protease that cleavages Scc1 during mitosis	Normal growth
*pds1-1*	Securin that inhibits Esp1 (protease) activity until metaphase	Normal growth
*pds5-1*	Recruited by cohesin, role unknown	Normal growth

### The Elg1 protein plays a role in sister chromatid cohesion

**Figure 5:**
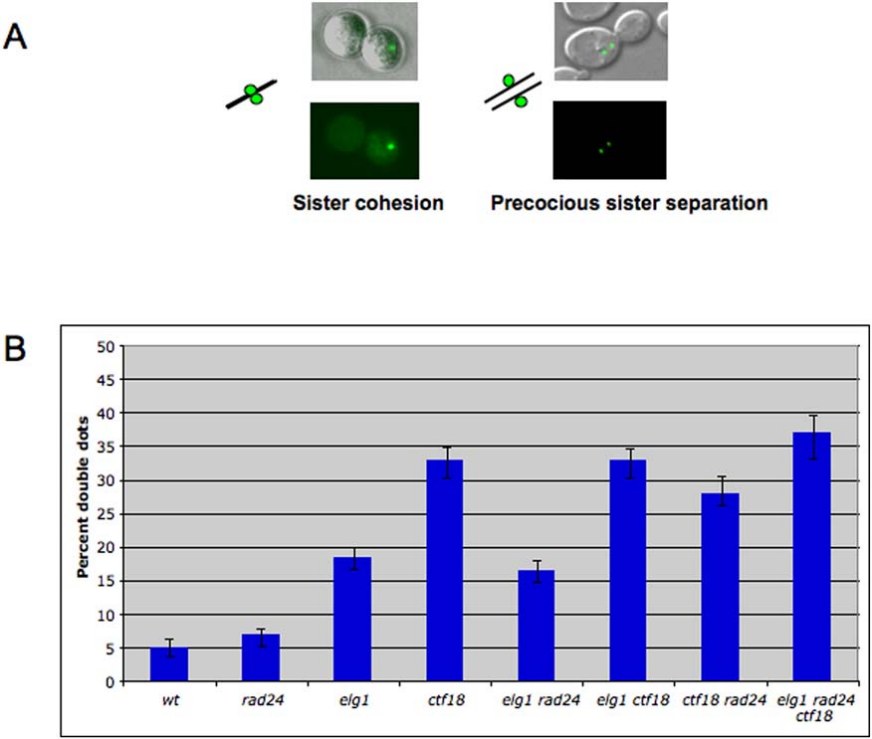
Elg1 and Ctf18, but not Rad24, play a role in sister chromatid cohesion. (A) Cohesion test. Isogenic strains carrying TetO tandem repeats on chromosome *V* were arrested in G2 with nocodazole and the percent of cells exhibiting two GFP dots (indicating separated sister chromatids) was counted. (B) Percentage of cells exhibiting precocious sister chromatid separation. At lest 400 cells were counted for each strain.

The results presented above suggest that Elg1 may have a direct role in sister chromatid cohesion. In order to directly measure this potential role we used a cohesion assay in which a *TetO* array is integrated approximately 40 kb from the centromere of chromosome *V*. These strains constitutively express a GFP-tagged TetR protein, which binds the arrays and can be visualized as a single GFP “dot”. In G2-arrested cells, lack of chromatid cohesion is seen as a “double dot” [13] (Figure 5A). Previous analysis, using a similar system, showed that the Elg1 homolog Ctf18 plays a role in sister chromatid cohesion [20]. Here we confirm these results and show that Elg1 has also a role in cohesion: whereas only 5% of wild type cells showed a “double dot” phenotype, *elg1* mutants showed this phenotype in 18.5% of the cells. Mutations in *CTF18* exhibited a stronger phenotype (33%). Interestingly, double mutants *elg1 ctf18* exhibit the same phenotype as the *ctf18* single, indicating epistatic interactions. In contrast, mutations in the gene encoding the third RFC-like protein, Rad24, had only a modest effect, if at all, in sister chromatid cohesion, and no aggravating phenotype was observed when the *rad24* mutation was combined with the *elg1* or *ctf18* mutations, or with the double mutant (Figure 5B). We thus conclude that both Ctf18 and Elg1 play a role in chromatid cohesion, with Ctf18 having a stronger contribution.

### Elg1 localizes to chromatin and plays a role in the recruitment of Ctf18

**Figure 6:**
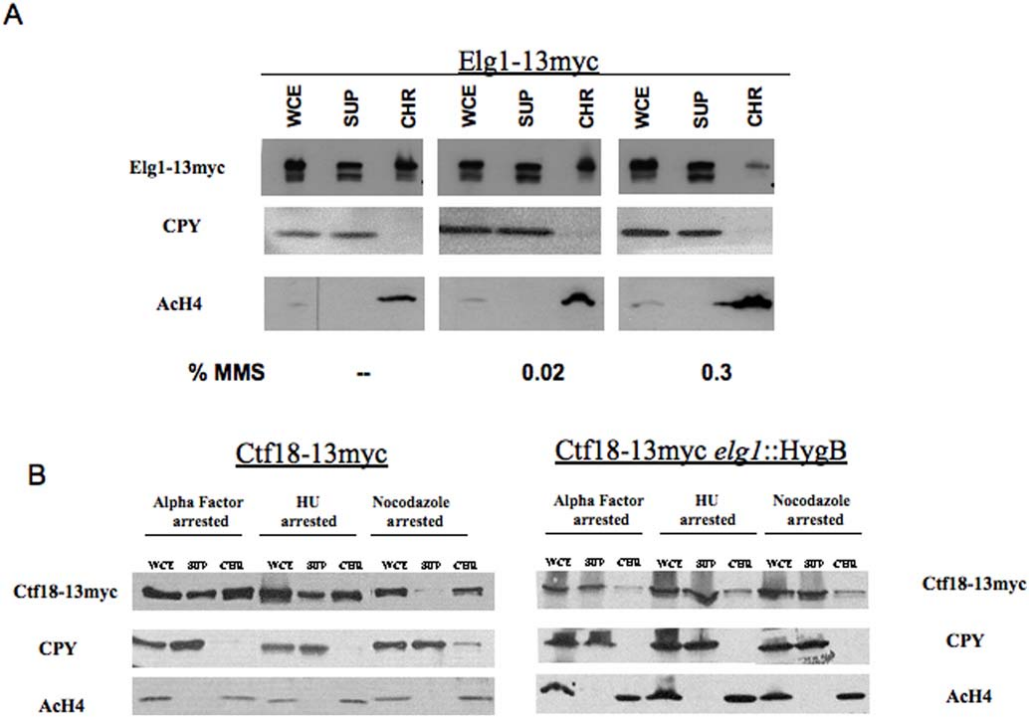
Elg1 and Ctf18 are present both at the chromatin and soluble fractions. Whole cell extracts from cells at mid-logarithmic growth phase were fractionated into a chromatin and a soluble fraction, and the presence of Myc- tagged Elg1 was followed with anti-Myc specific antibodies. As controls, anti-Carboxypeptidase Y and anti- Acetylated histone H4 antibodies were used. (A) Elg1 is present in both fractions, but its abundance decreases with DNA damage. Cells were untreated, or incubated in the presence of methyl methanesulfonate (MMS) at two different concentrations (0.02% and 0.3%). (B) Ctf18 is present in both fractions, and its localization in G2 depends on Elg1. Wild type or *elg1* cells were arrested with either alpha factor (G1), hydroxyurea (early S) or nocodazole (G2) before being fractionated.

To further examine the interactions between Elg1 and Ctf18, we analyzed the recruitment of these proteins to chromatin *in vivo*, using chromatin fractionation assays (Figure 6). Cells were spheroplasted, lysed, and the chromatin-containing fraction was separated from the soluble fraction. Appropriate fractionation was verified in all experiments by assessing the enrichment of acetylated histone H4 in the chromatin fraction and the enrichment of Carboxypeptidase Y (CPY) in the soluble fraction. Figure 6 shows that Elg1 was present both in the chromatin and in the soluble fraction. The protein was almost always seen as a double band; it is not clear yet what kind of modification or processing is responsible for this phenomenon. The amount of Elg1 at the chromatin fraction did not seem to fluctuate greatly during the cell cycle (data not shown) (Figure 6A). We also monitored the recruitment of Ctf18 to the chromatin fraction. Figure 6B shows that, similar to Elg1, Ctf18 is present both in the soluble fraction, as well as in the chromatin. Similar results were obtained in cycling cells (not shown), as well as in cells arrested in G1 with alpha factor or in early S with hydroxyurea. Interestingly, in cells arrested in G2 with nocodazole, most of the Ctf18 protein is present in the chromatin fraction. When the experiment was repeated in an *elg1* strain, the localization of Ctf18 changed, from being mainly in the chromatin, to being mainly at the soluble fraction (Figure 6B). Thus, Elg1 plays a role in determining Ctf18's localization to chromatin.

## Discussion

Elg1 and Ctf4 are two proteins that where found to interact genetically and physically with the replication fork. Elg1 binds the small subunits of the RFC complex [4] and, similarly to the RFC complex, is assumed to act during replication to load or unload the processivity clamp PCNA. Accordingly, physical contacts have been detected between these proteins and replication factors: Elg1 interacts with PCNA [[5] and data not shown], while Ctf4 interacts with polymerase α [14]. In a previous study we found that mutations in the *ELG1* and *CTF4* genes show a synthetic lethal (SL) interaction [4], suggesting that they jointly participate in an essential process in the cells. In order to uncover this essential process we performed a high copy number screen to find suppressors of the SL interaction. We found two proteins involved in sister chromatid cohesion: the cohesin subunit Scc1 and the cohesin loader complex subunit Scc2. These results suggest that the defective process common to these mutants is sister chromatid cohesion. Although it is well known that *ctf4* exhibits defects in sister chromatin cohesion [16], [17], [19], [20], Elg1 was not found previously to be involved in this process. The fact that high copy number plasmids carrying Scc1 or Scc2 are able to suppress the known phenotypes of the *elg1* mutant such as its sensitivity to MMS (Figure 3) indicates that *elg1* has a sister chromatid cohesion phenotype by itself, independently of its interactions with Ctf4. Using a direct sister chromatid cohesion assay, we show that *elg1* mutants indeed have a sister chromatid separation phenotype (Figure 5B), and have a 3.5-fold higher level of precocious separation, compared to wild type cells. This level is comparable to that observed in *bona fide* cohesion mutants, such as *chl1, bim1* or *kar3* [34].

Moreover, we also show that the activity of Elg1 becomes essential when there are defects in cohesin subunits or in its loading (Table 2 and Figure 4). The SL phenotype of *elg1* with cohesin components takes place in cells that have functional Ctf4 protein, again supporting a direct role for Elg1 in sister chromatid cohesion. Importantly, *elg1* exhibits synthetic genetic interactions with *scc2*, which was found to be important for cohesin loading [30] and with the subunits of the cohesin complex, but not with genes that are involved in late stages of cohesin maintainance or cohesin cleavage, such as *PDS1* [32], *ESP1* [31] and *PDS5* [33] (Table 2). These results point to an early role of Elg1 in sister chromatid cohesion, possibly during cohesion establishment or during DNA replication.

Our results show that out of the three non-essential RLCs, Ctf18 and Elg1 affect sister chromatid cohesion, whereas Rad24 seems to play at most a minor role in this process. This is consistent with the fact that the Rad24 RLC loads the 9-1-1 alternative clamp [11] whereas both Ctf18 and Elg1 have been shown to interact physically with PCNA, the replication clamp [3], [4], [19], [20]. Moreover, it has been recently found that *ctf18* mutants, as *elg1* mutants, are SL with *ctf4* [35].

This result is consistent with those presented in Figure 5, where we show that Elg1 affects cohesion via the same genetic pathway as Ctf18 (as the double mutant *elg1 ctf18* shows a similar phenotype to that of the single *ctf18* mutant). Moreover, we show that both proteins localize to chromatin, and that Elg1 is required in order to direct Ctf18 to the chromatin fraction (Figure 6B).

In previous studies, different interactions between *elg1* and *ctf18* were observed, depending on the phenotype examined. In response to DNA damage (MMS, UV light, hydroxyurea) the *elg1 ctf18* double mutant showed a higher sensitivity compared to the single mutants, suggesting that these proteins work in different pathways in the presence of DNA damage [4]. Contrasting phenotypes were seen in *elg1* and *ctf18* mutants of *S. pombe* [36] suggesting that the two RLCs act antagonistically to each other. Opposite effects were also observed at telomeres: *elg1* mutants exhibit elongated telomeres, whereas *ctf18* mutants have short telomeres [2], [8], [37]. Finally, it has recently been shown that mutations in either *ELG1* or *CTF18* have the same effect in suppressing the *hst3 hst4* mutant that contains hyperacetylated histone H3 at position K56 [38]. Interestingly, the same effect could be achieved by deletion of *CTF4*, consistent again with a model in which Elg1, Ctf18 and Ctf4 carry out related functions.

While this work was in progress, Maradeo and Skibbens [39] reported that mutations in *ELG1* can partially suppress the temperature sensitivity and the cohesion defects of an *ctf7/eco1-1* mutant, whereas Elg1 overexpression exacerbates its conditional growth defect. Ctf7 is a pivotal sister chromatid cohesion factor that was found to be important for cohesion establishment [25], [26] and for sister chromatid cohesion in response to DNA damage [40]. The reported genetic interaction between *ELG1* and *ctf7* strengthen our characterization of Elg1 as a cohesion factor. We further extend these conclusions by analyzing the interactions between *elg1* and all known cohesion factors. In addition, we show that Elg1 plays a possible role in recruiting the Ctf18 RLC to the chromatin, thus providing a mechanistic explanation for the epistatic genetic interactions between *elg1* and *ctf18* in sister chromatid cohesion. Consistent with our results (Figure 5B), no genetic interactions could be detected between *ctf7/eco1-1* and the third RLC, containing Rad24 [39].

The physical interactions of Ctf18 and Elg1 with the small RFC subunits and with PCNA and the genetic interactions with *CTF4*, a gene encoding a Polymerase α -interacting protein, suggest a model in which the two RLC subunits and Ctf4 mediate the interaction between the DNA replication machinery and the loading of cohesin. Cohesin is loaded onto DNA at the end of G1 [30]. Thus, during DNA replication, it is expected that the progressing fork would encounter cohesin molecules on its path. A possible model of action of the RLCs and of Ctf4 would be that passage through a cohesin loop may require momentary unloading, then re-loading, of PCNA. This would explain the physical and genetic interactions of these proteins with DNA replication components, as well as with cohesin and its associated proteins.

## Materials and Methods

### Yeast strains and plasmids

A list of all strains used in this paper is presented as Table 3. Plasmids pSB418 (*URA3-ELG1-ADE3*) and pSBA419 (*LEU2-ELG1-ADE3*) have been described [41]. High copy number *URA3*-marked and *LEU2*-marked plasmids pSCC1, pSCC2, pSMC1, pSMC3 and pELG1 were created by cloning the respective genes in YEplac181 (*LEU2*) or YEplac195 (*URA3*).

**Table 3:** List of strains used in this study.

Strain	Genotype	Reference
MK166	*MATα lys2:: Ty1Sup ade2-1(o) can1-100(o) ura3-52 leu2-3, 112 his3del200 trp1del901 HIS3 :: lys2 :: ura3 his4 :: TRP1 :: his4*	[42]
BY7471*elg1*	*MATa elg1 :: KanMX his3-1 leu2-0 met15-0 ura3-0*	[45]
BY7471*ctf4*	*MATa ctf4 :: KanMX his3-1 leu2-0 met15-0 ura3-0*	[45]
*MK1001*	*MATa elg1 ::KanMX ctf4:KanMX lys2:: LTR ade2-1(o) ade3 del can1-100(o) ura3-52 leu2-3, 112 his3del200/pSBA419 (LEU2-ELG1-ADE3)*	[4]
*DD282*	*MATa his3_1 leu2_0 met15_0 ura3_0 pep4Δ::KAN ELG1::13myc-his5.*	[5]
6745	*MATα ade2-1 trp1-1 can1-100 leu2-3,112 his3-11,15 ura3::3xURA3tetO112 leu2::LEU2 tetR-GFP*	[46]
6745*elg1*	*MATα elg1::LEU2*	This study
6745*ctf18*	*MATα ctf18::HygB*	This study
6745*rad24*	*MATα rad24::KanMX*	This study
6745*rad24ctf18*	*MATα rad24::KanMX ctf18::HygB*	This study
6745*elg1 rad24*	*MATa elg1 :: LEU2 rad24::KanMX*	This study
6745*elg1 ctf18*	*MATa elg1 :: LEU2 ctf18 :: HygB*	This study
6745*elg1 ctf18 rad24*	*MATa elg1 :: LEU2 ctf18 :: HygB rad24::KanMX*	This study
W303A*scc1-73*	*MATa ura3-1 ade2-1 trp1-1 can1-100 leu2-3,112 his3-11,15 scc1-73*	[46]
W303A*smc1-259*	*MATa ura3-1 ade2-1 trp1-1 can1-100 leu2-3,112 his3-11,15smc1 -259*	[46]
W303A*smc3-42*	*MATa ura3-1 ade2-1 trp1-1 can1-100 leu2-3,112 his3-11,15 smc3-42*	[46]
W303A*scc3-1*	*MATa ura3-1 ade2-1 trp1-1 can1-100 leu2-3,112 his3-11,15 scc3-1*	[46]
W303A *scc2-4*	*MATa ura3-1 ade2-1 trp1-1 can1-100 leu2-3,112 his3-11,15 scc2-4*	[30]
W303A *scc4-4*	*MATa ura3-1 ade2-1 trp1-1 can1-100 leu2-3,112 his3-11,15 scc4-4*	[30]
SY96	*MATa esp1-1 leu2-3, 112 ura3-52 lys2 can1*	B. Byers collection
VG986-5B	*Mata trp1 ura3 bar1 pds5-1*	[33]
RA 2806-2b	*MATa pds1-38-HA::URA3 ESP1-Myc::TRP1 ura3-1 ade2-1 trp1-1 can1-100 leu2-3,112 his3-11,15*	[47]

### Genetic screen

Strain MK1001 (*elg1 ctf4 ura3 ade2 ade3 leu2/pSBA419*) was transformed with a high copy number *URA3* library as described [41]. After re-streaking colonies suspected of carrying suppressor plasmids, these were isolated and re-transformed into fresh MK1001 cultures. The plasmids in positive transformed colonies were sequenced.

### Recombination assay

The rate of recombination of wild type, *elg1* and *ctf4* mutants carrying various plasmids was measured as described [4]in derivatives of strain MK166 [42].

### Chromatin fractionation assay

Logarithmically growing cultures were arrested in G1 with α factor, at the beginning of S-phase with hydroxyurea or in G2 with nocodazole and incubated for one hour before being cropped by centrifugation [43]. Fractionation was carried out as in [44]. In brief, cells were incubated for 10 min at 37°C with 20 µg/µl of zymolase T-100, then lysed with 0.25% Triton X-100. Lysates were separated to supernatant and chromatin fractions by sucrose gradient. Whole cell extracts (WCE), supernatant (Sup) and chromatin pellets (CHR) were subjected to SDS-page Western blot using anti myc (SC-789, Santa Cruz Biotechnology), anti acetylated histone H4 (SC-06-946) or anti-CPY (SC-0998) antibodies. 30–50 µg of total proteins were loaded in each lane. Acetylated histone H4 serves as a loading control as well as marker for chromatin fraction and CPY serves as a marker for the soluble fraction.

### Two dot assay

This experiment was carried out as described [30]. At least 400 cells were counted for each strain.
